# Performance of Endobronchial Ultrasound-Guided Cryobiopsy in Diagnosing Thoracic Disorders and Its Role in Next-Generation Sequencing for Non-Small-Cell Lung Cancer

**DOI:** 10.1155/pm/3522554

**Published:** 2025-08-28

**Authors:** Chun Ian Soo, Sze Shyang Kho, Wai Ling Leong, Shinye Eng, Diana Bee-Lan Ong, Seow Fan Chiew, Tak Kuan Chow, Hazwan Amzar Khairul Annuar, Chee Kuan Wong, Chong Kin Liam

**Affiliations:** ^1^Department of Medicine, Faculty of Medicine, Universiti Malaya, Kuala Lumpur, Malaysia; ^2^Department of Medicine, Sarawak General Hospital, Kuching, Sarawak, Malaysia; ^3^Department of Biomedical Imaging, Universiti Malaya Research Imaging Centre, Universiti Malaya, Kuala Lumpur, Malaysia; ^4^Department of Pathology, Universiti Malaya, Kuala Lumpur, Malaysia

## Abstract

**Background:** Endobronchial ultrasound-guided transbronchial needle aspiration (EBUS-TBNA) is an established procedure for diagnosing thoracic diseases and staging of lung cancers. However, some limitations of cytology specimens from EBUS-TBNA include small sample size, low tumour cellularity, necrosis and specimen contamination. Endobronchial ultrasound-guided transbronchial mediastinal cryobiopsy (EBUS-TBMC) is a promising alternative that provides a larger histology specimen which may improve diagnostic accuracy and molecular testing. This study is aimed at evaluating the benefits of EBUS-TBMC over EBUS-TBNA, focusing on improving next-generation sequencing (NGS) success rates, and assessing its efficacy and safety in a real-world setting.

**Methods:** Data from 203 patients (99 underwent EBUS-TBNA and 104 underwent EBUS-TBMC) were retrospectively traced and analysed using descriptive statistics.

**Results:** The overall diagnostic yield was significantly higher for EBUS-TBMC (90.38%) than that for EBUS-TBNA (67.68%; *p* < 0.001). For heterogeneous lesions, the diagnostic yield was 92.31% for EBUS-TBMC and 69.44% for EBUS-TBNA (*p* = 0.011). For non-small-cell lung cancer (NSCLC), EBUS-TBMC specimens demonstrated higher overall tumour cellularity (65% vs. 30%; *p* < 0.001) and better success in detecting driver alterations through NGS (85.36% vs. 61.90%; *p* = 0.035). The median procedure duration was shorter for EBUS-TBMC (22 vs. 32 min; *p* < 0.001), and the complication rates were comparable between the two techniques. These findings suggest that EBUS-TBMC offers additional diagnostic advantages over EBUS-TBNA for heterogeneous lesions and significantly facilitates the acquisition of cell-rich specimens for NGS testing.

**Conclusion:** EBUS-TBMC increases the overall diagnostic yield of mediastinal diseases. EBUS-TBMC provides cell-rich histology specimens with high tumour content, facilitating NGS testing in the management of NSCLC.

## 1. Introduction

Endobronchial ultrasound (EBUS)-guided transbronchial needle aspiration (EBUS-TBNA) is a well-established, minimally invasive procedure for the diagnosis and staging of lung cancer and the evaluation of mediastinal disease [1–3]. However, EBUS-TBNA only provides cytology specimens or small core tissues, which often require specialised processing, such as cell block preparation, to enhance diagnostic accuracy. One of the main challenges frequently encountered with EBUS-TBNA includes nondiagnostic specimens, which may result from a small specimen size, sampling of nodal micrometastasis, or contamination with blood or bronchial cells [4, 5]. Another commonly encountered challenge is the sampling of necrotic lesions, which often results in low cellularity. Although a morphological diagnosis may still be possible, the limited number of tumour cells can hinder ancillary testing and molecular analysis. This is particularly important in molecular targeted therapy for non-small-cell lung cancer (NSCLC).

Endobronchial ultrasound-guided transbronchial mediastinal cryobiopsy (EBUS-TBMC) has recently gained popularity. This method offers the advantage of obtaining histological specimens, allowing the acquisition of larger tissue specimens while preserving the histological architecture. EBUS-TBMC has the potential to increase the diagnostic accuracy of EBUS-guided mediastinal biopsies, especially for uncommon malignancies, benign disorders and lymphomas [6–8]. In the diagnosis of NSCLC, although studies indicate that EBUS-TBMC may not significantly improve the diagnostic yield compared to conventional EBUS-TBNA, EBUS-TBMC specimens demonstrate a higher concentration of tumour cells and may improve advanced molecular testing, such as next-generation sequencing (NGS) [8]. Owing to the lack of real-world data, whether EBUS-TBMC is superior for obtaining adequate specimens for detecting driver alterations through NGS remains uncertain, particularly in heterogeneous lesions identified on preprocedural computed tomography (CT) with a high likelihood of tumour necrosis. To address these knowledge gaps, we aimed to evaluate the potential benefits of EBUS-TBMC over conventional EBUS-TBNA for heterogeneous lesions and the adequacy for NGS testing in NSCLC.

## 2. Methods

### 2.1. Study Design and Patient Recruitment

**Figure 1:**
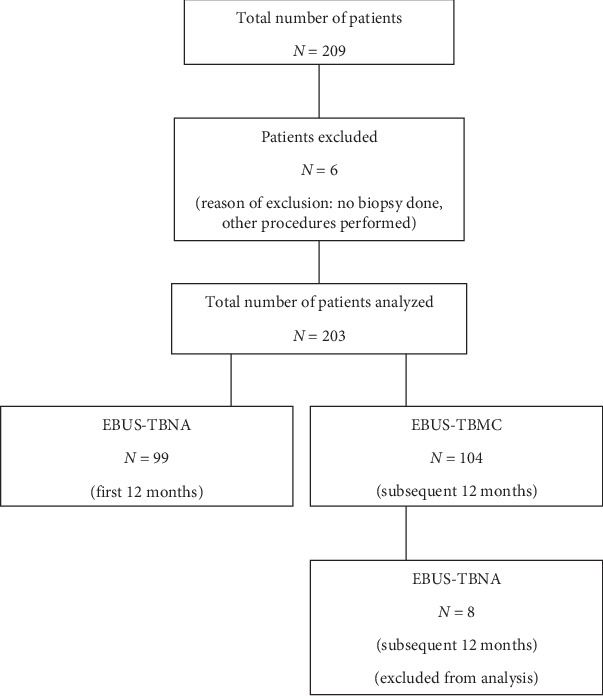
Flow diagram for patient selection. EBUS-TBNA: endobronchial ultrasound–transbronchial needle aspiration; EBUS-TBMC: endobronchial ultrasound–transbronchial mediastinal cryobiopsy.

Our institution adopted the use of EBUS-TBMC in 2023. Data of patients who underwent EBUS-TBNA in 2022 and EBUS-TBMC in 2023 were retrospectively retrieved from electronic medical records for analysis. The indications for EBUS-TBNA and EBUS-TBMC were diagnosis of mediastinal diseases and diagnosis and staging of lung cancer. Data from patients who underwent EBUS without sampling and EBUS-TBNA following EBUS-TBMC in 2023 were excluded from the analysis. The flow diagram for patient selection is shown in (Figure 1).

### 2.2. Review of Imaging

Target lesions on the patients' CT images were reviewed by two general radiologists with more than 5 years of experience. Radiological assessment for heterogeneity was based on whether the lesion exhibited visual nonuniformity and whether two selected regions of interest in the lesion demonstrated a difference in the highest and lowest attenuation of more than 20 Hounsfield units in a contrast enhanced CT [9]. The heterogeneity of the lesion was also assessed by an EBUS bronchoscopist using B-mode imaging according to the standard EBUS image classification system [10]. Both the radiologists and EBUS bronchoscopist were blinded to the clinical details and results of each other's assessments. In a pilot study involving 20 patients to determine the degree of agreement between the radiologists in categorising heterogeneity in the targeted lesions, the overall Cohen's kappa (*κ*) value for the study was 0.905 (95% CI, 0.300–0.886), *p* < 0.001 (Supporting Information 3: Table S2). A lesion was classified as truly heterogeneous only when all three evaluators' findings were consistent.

### 2.3. Procedure

EBUS-TBNA and EBUS-TBMC were performed by a European Respiratory Society-accredited EBUS bronchoscopist with 8 years of experience. EBUS-TBNA was performed according to established guidelines [3]. Fujifilm EB-530US (Fujifilm, Tokyo, Japan) EBUS was used for all procedures. The choice of the TBNA needle was left to the discretion of the bronchoscopist based on the size and location of the target lesion. The EchoTip ProCore Endobronchial HD biopsy needle (Cook Medical, Bloomington, IN, United States) of either 22- or 25-gauge sizes was used, whereas the ViziShot 2 (NA-401SX-4021; Olympus Ltd, Tokyo, Japan) was used in cases requiring a 21- or 19-gauge needle. The EBUS procedure was usually performed under conscious sedation with intravenous midazolam (0.01–0.1 mg/kg) and small boluses of intravenous fentanyl of 25 mcg until the desired effect is achieved [11]. Total intravenous anaesthesia was used in challenging cases in which longer procedure times were anticipated.

For EBUS-TBNA, the use of suction and additional TBNAs was left to the discretion of the bronchoscopist when the volume of the specimen obtained was macroscopically examined. During sample preparation, one drop of the aspirated specimen was gently smeared onto a slide, air-dried and fixed in ethanol. Additional specimens were air-flushed from the needle into a Cytolyt solution, cytospined and processed into a cell block. The EBUS-TBMC procedure followed previously described steps [12]. An ultraslim 1.1-mm cryoprobe (ERBE Medizintechnik, Tübingen, Germany) was used for cryobiopsy. The duration of cryoactivation was left to the operator's discretion based on the size of each tissue macroscopically examined. The cryobiopsy specimens were thawed in room temperature saline and transferred into a container with formalin. A dose of prophylactic intravenous antibiotics (intravenous ceftriaxone 2 g) was administered to the patient after the procedure when sampling was performed on heterogeneous lesions. The degree of bleeding was classified into mild (spontaneous cessation without intervention) and moderate (requiring suction for more than 1 min or the instillation of cold saline or a vasoactive substance) [13]. Desaturation during the procedure was defined as a drop in oxygen saturation by pulse oximetry (SpO_2_) of more than 4% or SpO_2_ of less than 90% lasting for more than 1 min [14]. Patients were followed up for 6 months to monitor postprocedure complications.

### 2.4. Definitions

#### 2.4.1. Cryoactivation Time

The total cryoactivation time, representing the cumulative time of each cryoactivation cycle, was documented for every TBMC procedure. The average cryoactivation time per cryopass was determined by dividing the total cryoactivation time by the corresponding number of cryopasses.

#### 2.4.2. Representative Specimen and Diagnostic Yield

For cytological specimens involving lymph nodes, the EBUS-TBNA specimen is considered representative if it demonstrated the presence of a germinal centre, at least 100 lymphocytes per field at ×100 magnification, and anthracotic pigment-laden macrophages [15]. The EBUS-TBMC specimen was considered representative with the presence of lymphoid tissue and macrophages with tangible bodies [16]. Overall, a specimen obtained by EBUS-TBNA or EBUS-TBMC is deemed conclusive if a cytological or histopathological examination can provide a definitive diagnosis. For nonpathological nodes, further radiological surveillance for at least 6 months was conducted to confirm clinical stability [6, 8, 17]. The presence of atypical cells or suspicion of malignancy was categorised as inconclusive in terms of diagnostic yield. NSCLC and small-cell lung cancer were categorised under common tumour. Otherwise, the other metastatic tumours were put in the same group with lymphoma. Benign disorders consisted of sarcoidosis, tuberculosis and reactive lymph node.

#### 2.4.3. NGS Testing

NGS testing was conducted on specimens showing a diagnosis of NSCLC. NGS testing incorporated both DNA- and RNA-based variants using the Oncomine precision assay GX (Thermo Fisher Scientific, Waltham, MA, United States). The panel detected hotspot mutations (substitutions, insertions and deletions), copy number variations and gene fusions across 50 cancer driver genes (Table 1).

### 2.5. Statistical Analysis

Data analysis was performed using the SPSS software (Version 29; Chicago, IL, United States). All data were checked for normality using the Shapiro–Wilk test. Mean ± standard deviation was used for normally distributed data, while nonnormally distributed variables were presented as median and interquartile range (IQR). Categorical data were summarised as absolute numbers and percentages and analysed using Pearson's chi-square test or Fisher's exact test. For comparisons between groups, independent sample *t*-tests were used for normally distributed variables, and Mann–Whitney test was used for nonnormally distributed variables. Statistical significance was set at *p* < 0.05.

### 2.6. IRB Approval

The study protocol was approved by the Medical Research and Ethics Committee of the Universiti Malaya Medical Centre (MREC ID NO: 2024418-13645), approval date 9 May 2024. The study followed the International Conference on Harmonisation Guidelines for Good Clinical Practice and the principles of the Declaration of Helsinki.

## 3. Results

### 3.1. Baseline Demographics and Procedural and Lesion Characteristics

A total of 203 patients were included in the study. Then, 99 patients underwent EBUS-TBNA, and 104 underwent EBUS-TBMC. The patients' basic demographics and EBUS-TBNA and EBUS-TBMC details are shown in Table 2. The median age for the TBNA and TBMC cohorts was 65.0 (IQR 57.00–71.00) years and 64.0 (IQR 56.00–69.75) years, respectively. In both cohorts, two-thirds of the patients were male and the majority (approximately 54%) were nonsmokers. Conscious sedation was used in 78.79% and 67.31% of EBUS-TBNA and EBUS-TBMC procedures, respectively. The 22G needle was most commonly used (70.71%), with 5 cmH20 suction applied in 57.58% of EBUS-TBNA procedures. A median of four (IQR 4.00–5.25) EBUS-TBNA passes were performed for each lesion. For EBUS-TBMC, a 22G needle was used for biopsy track creation in 86.54% of cases. On average, four (IQR 3–4) EBUS-TBMC passes were performed for each lesion. The median cumulative cryoactivation time was 24 s (IQR 18–32) and the average cryoactivation time was 8 s (IQR 6–8). A total of 134 EBUS-TBNA (for 36 masses and 98 lymph nodes) and 140 EBUS-TBMC (for 38 masses and 102 lymph nodes) procedures were performed. In the TBNA group, the mean size widest diameter of the masses was 27.49 ± 12.20 mm, and the short axis diameter on CT scan of the lymph nodes measured a median of 14.4 (IQR 10.15–20.13) mm. In the TBMC group, the mean size widest diameter of the masses was 26.37 ± 9.97 mm while the median size short axis diameter on CT scan of the lymph nodes was 13.95 (IQR 11.00–18.15) mm. Heterogeneous lesions were identified in 36.57% and 32.14% of patients in the EBUS-TBNA and EBUS-TBMC cohorts, respectively.

### 3.2. Diagnostic Yield

The overall diagnostic yield of EBUS-TBMC was 90.38% (94 of 104 patients), compared with that of 67.68% (67 of 99 patients) for EBUS-TBNA (*p* < 0.001) (Table 3). However, the diagnostic yield for common tumours was not significantly different between the two techniques. The diagnostic yields of EBUS-TBNA and EBUS-TBMC for other metastatic malignancies were 70.00% (seven of 10 cases) and 95.45% (21 of 22 cases), respectively (*p* = 0.04). In benign disorders, the diagnostic yield for sarcoidosis was 50% for EBUS-TBNA (one of two cases) versus all four cases (100%) for EBUS-TBMC (*p* = 0.33) and the diagnostic yield for tuberculosis was 62.50% (five of eight cases) for EBUS-TBNA and 100% (16 cases) for EBUS-TBMC (*p* = 0.03). Interestingly, in heterogeneous lesions, the diagnostic yield of EBUS-TBMC was significantly higher than that of EBUS-TBNA (92.31% (36 of 39 patients) vs. 69.44% (25 of 36 patients) (*p* = 0.011)).

For patients in whom the EBUS procedures were nondiagnostic, the diagnosis was made using other procedures (transbronchial lung biopsy, CT-guided percutaneous needle biopsy, mediastinoscopy or video-assisted thoracic surgery). The results are available in Supporting Information 1: Table S1.

### 3.3. NGS

**Figure 2:**
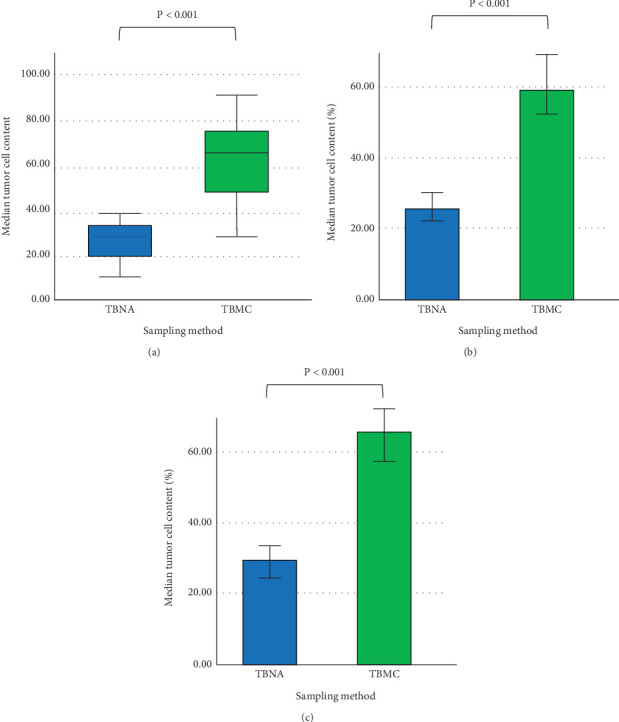
(a) Box plot comparison of overall tumour cells from TBNA versus TBMC. (b) Box plot comparison of tumour cells from TBNA versus TBMC in nonheterogeneous lesions. (c) Box plot comparison of tumour cells from TBNA versus TBMC in heterogeneous lesions. TBNA: transbronchial needle aspiration; TBMC: transbronchial mediastinal cryobiopsy.

Overall, of the 203 cases, 83 (42 (50.60%) from EBUS-TBNA and 41 (49.40%) from EBUS-TBMC) cases of NSCLC required molecular testing using NGS. The median cellularity of EBUS-TBMC specimens was 65% (IQR 50–75) tumour cells compared with a median of 30% (IQR 20–35) of EBUS-TBNA specimens (*p* < 0.001). Subgroup analysis revealed that the tumour content of specimens obtained by TBMC was significantly higher than that obtained by TBNA, irrespective of lesion heterogeneity (Figure 2). In nonheterogeneous lesions, the mean tumour content was 25.38% (±1.94) for TBNA specimens compared with 58.91% (±3.30) for TBMC specimens (*p* < 0.001). Similarly, in heterogeneous lesions, the mean tumour content was 28.75% (±8.66) for TBNA specimens compared with 66.18% (±17.28) for TBMC specimens (*p* < 0.001). The proportion of NSCLC cases with successful detection of driver alterations by NGS was significantly higher (85.36% in the TBMC group compared to 61.90% in the TBNA group, *p* = 0.035).

In the TBNA cohort, an insufficient number of tumour cells led to limited biomarker testing for PD-L1 by immunohistochemical (IHC) staining and sequential testing for *EGFR* mutation by real-time polymerase chain reaction (RT-PCR), *ALK* rearrangement by IHC staining and *ROS1* fusion by fluorescent in situ hybridisation (FISH)) in nine patients (21.43%). Four patients (9.52%) underwent only RT-PCR testing for *EGFR* mutation, while biomarker testing could not be performed in three patients (7.14%). In contrast, all six patients (14.63%) in the TBMC group in whom NGS could not be performed because of tumour content insufficiency had adequate tumour cells for limited biomarker testing (Supporting Information 2: Figure S1).

### 3.4. Procedure Time and Complications

The median overall duration of EBUS-TBMC was 22 min (IQR 18–32) versus 32 min (IQR 27–37) for EBUS-TBNA (*p* < 0.001). Mild bleeding post biopsy (21.21% in the EBUS-TBNA group vs. 22.11% in the EBUS-TBMC group) was the most common complication. Both cohorts had bleeding in 3.03% (three cases) of cases, requiring extended scope compression at the puncture site. One case of self-limiting pneumomediastinum was recorded after EBUS-TBMC. No other major complications were observed.

## 4. Discussion

### 4.1. Diagnostic Yield

EBUS-TBNA is a safe and well-established procedure for the diagnosis and staging of lung cancer [3]. However, in real-world practice, EBUS-TBNA has certain limitations. Inadequate sample acquisition remains a common shortcoming faced by bronchoscopists [4, 5]. The use of EBUS-TBMC has attracted considerable interest because of its potential to provide larger specimens and enhance diagnostic yield.

**Figure 3:**
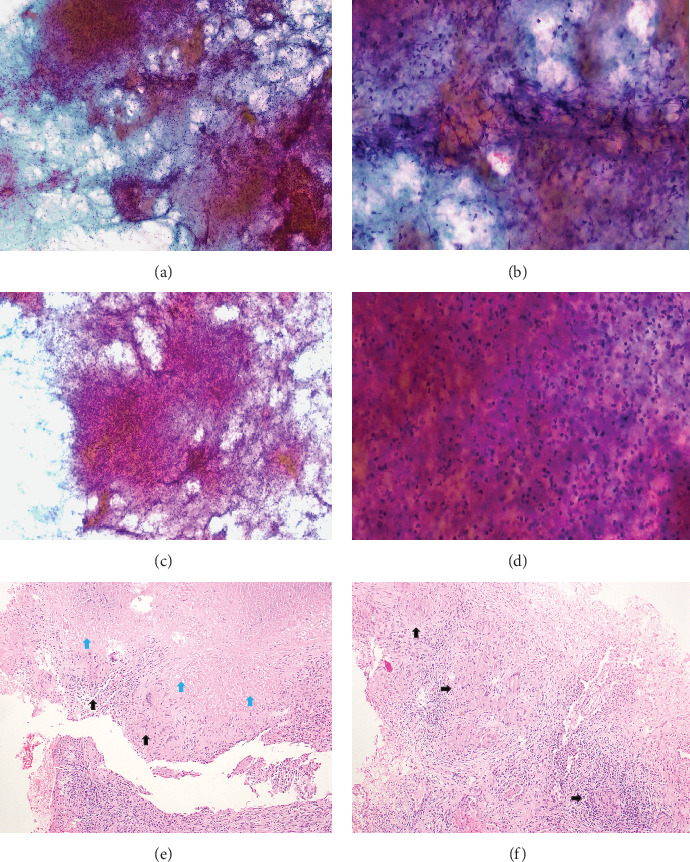
(a–c) Degenerated cellular debris with crushed cells: (a, c) papanicolaou (PAP) ×10 and (b) PAP ×20. (d) Degenerate cellular debris with crushed cells and background of abundant neutrophils PAP × 40. (e, f) Aggregates of slender-shaped macrophages forming epithelioid granulomas, with central necrosis. Black arrow: granuloma, blue arrow: necrosis. Hematoxylin and eosin (H&E) ×10.

**Figure 4:**
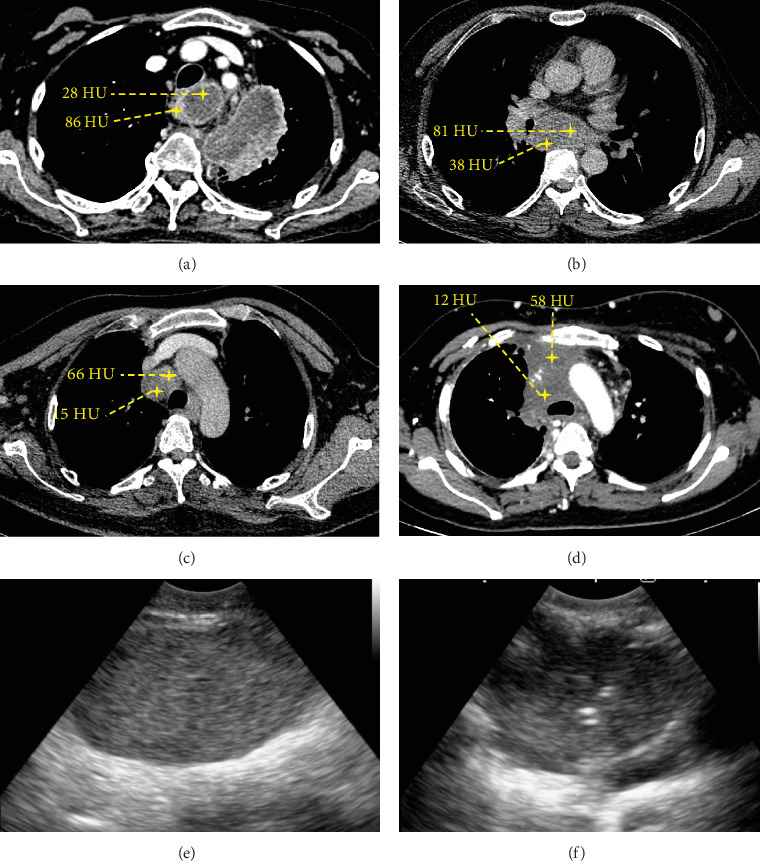
Representative cases of heterogenous lesions in this study. These images show two areas with a Hounsfield unit difference of at least 20, indicating lesion heterogeneity. (a) Heterogenous left upper paratracheal lymph nodes (Station 2L). (b) Heterogenous subcarinal lymph node (Station 7). (c) Heterogenous right upper paratracheal lymph nodes (Station 2R). (d) Heterogenous matted anterior mediastinal lymph node. (e) Homogenous appearance of a mediastinal lymph node (Station 4L) visualized using B-mode EBUS. (f) Heterogenous appearance of a mediastinal lymph node (Station 4R) with a cryoprobe in situ visualised using B-mode EBUS. TBNA: transbronchial needle aspiration; TBMC: transbronchial mediastinal cryobiopsy.

Two meta-analyses demonstrated that EBUS-TBMC has an overall higher pooled diagnostic yield than EBUS-TBNA, especially for benign and lymphoproliferative disorders [18, 19]. In the diagnosis of lung cancer, the pooled analysis showed no statistically significant difference between EBUS-TBMC and EBUS-TBNA (96% vs. 92.5%: OR of 1.72, 95% CI: 0.80–3.70; *p* = 0.16) [19]. Our findings are consistent with those of the existing literature. Although the overall diagnostic yield of EBUS-TBMC was higher than that of EBUS-TBNA, a significant difference was observed for other metastatic malignancies and tuberculosis. For NSCLC, the diagnostic yield of EBUS-TBNA was not inferior to that of EBUS-TBMC. However, EBUS-TBMC demonstrated a higher diagnostic yield when the lesions were heterogeneous on CT. Lesions exhibiting imaging heterogeneity are often associated with increased angiogenesis and a high prevalence of malignancy. However, heterogeneous lesions may show necrosis and reduced cellularity [20–22] (Figure 3). Therefore, positron emission tomography–CT fusion imaging has been employed to identify regions with viable tumours so that necrotic areas can be avoided during biopsy, thereby enhancing the likelihood of obtaining diagnostically valuable specimens [23].  Figure 4 illustrates a few representative cases of heterogeneous lesions that were sampled and included in the analysis in this study. The rationale behind the higher diagnostic yield of EBUS-TBMC in heterogeneous lesions is that TBMC specimens are typically larger, more cellular, and exhibit better-preserved architecture, facilitating a more thorough histological evaluation than cytology specimens.

### 4.2. Subtyping of NSCLC

The management of NSCLC extends beyond just obtaining a morphological diagnosis. Often, further ancillary testing is required, which can be challenging because EBUS-TBNA provides only cytological or small-core specimens. High-quality specimens with sufficient cellular content are essential to address the need for accurate subtyping and differentiation between primary and metastatic diseases. Accurate subtyping of NSCLC is essential for therapeutic selection. Poorly differentiated carcinomas with adenocarcinoma immunoprofiles have survival outcomes similar to those of morphologically defined adenocarcinomas, highlighting their importance [24]. Subtyping NSCLC with mixed morphology (e.g. adenosquamous) and poorly differentiated or undifferentiated features can be highly challenging, often requiring further IHC testing that may consume a substantial portion of the available tissue material. In addition, an adequate specimen is crucial for IHC testing to identify the primary site of the disease, particularly in metachronous tumours, as this influences treatment strategies.

### 4.3. Molecular Testing and NGS in NSCLC

Earlier studies have shown that four EBUS-TBNA passes yield over 90% success in limited molecular profiling (*EGFR*, *KRAS* and *ALK* alterations) [25, 26]. Over the past decade, major therapeutic advancements have transformed the NSCLC treatment landscape. The range of targeted therapies continues to expand, shifting the paradigm from limited molecular testing to comprehensive driver alteration detection through NGS, which can assess hundreds of biomarkers. For NGS testing, success rates with cytology specimens varied from 46.0% to 95.3%, whereas four core tissue specimens achieved an 83.3% success rate with 73.8% sensitivity and 61.5% specificity [27, 28]. A meta-analysis of 21 studies reported an 86.5% pooled adequacy rate of EBUS-TBNA specimens (95% CI: 80.9%–91.4%) for NGS [29]. In our study, the success rate of NGS testing was significantly higher with EBUS-TBMC, regardless of lesion heterogeneity. The larger specimen size from EBUS-TBMC has the advantage of higher tumour cell content, which played a significant role in these findings [30]. The findings of our study align with those of a previous randomised controlled trial demonstrating successful NGS testing rates of 95%–97% with EBUS-TBMC, compared to 74%–79% with EBUS-TBNA [8].

### 4.4. Rapid On-Site Evaluation (ROSE) and the Proposed Extended Algorithm

**Figure 5:**
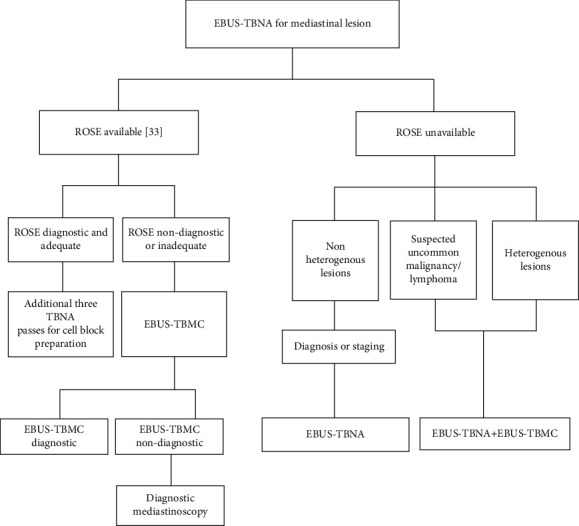
Proposed algorithm for EBUS. ROSE: rapid on-site evaluation; EBUS-TBNA: endobronchial ultrasound–transbronchial needle aspiration; EBUS-TBMC: endobronchial ultrasound–transbronchial mediastinal cryobiopsy.

When dealing with NSCLC, the importance of ROSE during EBUS-TBNA is to ensure that sufficient high-quality cytology material is triaged to the cell block to obtain adequate neoplastic DNA and RNA for specific gene alteration (mutation, translocation or amplification) testing [31]. Specimen contamination with blood and benign bronchial or cartilage cells can affect interpretation and reduce DNA/RNA extraction for molecular analysis [4, 27, 32]. Based on our study, in which ROSE was unavailable, we propose a new diagnostic algorithm for EBUS-TBMC (Figure 5). In nondiagnostic ROSE cases, EBUS-TBMC can supplement EBUS-TBNA, as suggested by Maturu et al. [33]. Where ROSE is unavailable, EBUS-TBMC may be considered, balancing its benefits and drawbacks. Although EBUS-TBNA remains the standard for the diagnosis and staging of lung cancer, EBUS-TBMC is a valuable alternative for improving the sample adequacy for comprehensive molecular profiling by NGS, especially for heterogeneous lesions.

### 4.5. Limitations and Strength of This Study

This study had several important limitations. The results were obtained from a single centre. The retrospective design introduces the potential for recall and selection biases. However, this study is deemed important as real-world data on NGS adequacy from EBUS-TBMC are lacking. At the time of this study, there was no standardised procedural protocol for EBUS-TBMC. The overall number of cryobiopsy passes and activation time were slightly higher than those reported in the current literature [34], which could be attributed to the fact that one-third of the lesions were heterogeneous, prompting the bronchoscopist to obtain larger specimens to optimise the overall diagnostic yield. The second limitation of this study is the use of CT imaging for assessment instead of PET-CT prior to the EBUS procedures. This was due to the limited availability of PET-CT services at our centre, resulting in long waiting times, making its routine use impractical.

Another limitation was the radiological assessment of heterogeneous lesions. Heterogeneity can indicate malignancy, areas of low cellularity or extensive necrosis. To minimise interobserver variability, all three evaluators were blinded during lesion classification. Review bias from unblinded pathologists favouring specimens from EBUS-TBMC over EBUS-TBNA has been highlighted by Poletti et al. [35]. Hence, to mitigate procedural bias in this study, EBUS-TBNA results after EBUS-TBMC in 2023 were excluded from the analysis. Ideally, a comparison of NGS suitability between TBNA and TBMC should be conducted in all cases; however, this was not feasible because of the retrospective study design.

## 5. Conclusions

EBUS-TBMC delivers histological specimens with greater tumour content, improving the success rate of detecting driver alterations using NGS in the management of NSCLC. As the demand for cell-rich specimens grows with the expanding list of recommended genetic alterations, large-scale prospective comparative studies focusing on molecular and cost analyses are eagerly awaited.

## Figures and Tables

**Table 1:** Cancer-related genes analysed with next-generation sequencing using Oncomine precision assay GX.

**Gene assay for the detection of DNA sequence variants**
AKT1, AKT2, AKT3, ALK, AR, ARAF, BRAF, CDK4, CDKN2A, CHEK2, CTNNB1, EGFR, ERBB2, ERBB3, ERBB4, ESR1, FGFR1, FGFR2, FGFR3, FGFR4, FLT3, GNA11, GNAQ, GNAS, HRAS, IDH1, IDH2, KIT, KRAS, MAP2K1, MAP2K2, MET, MTOR, NRAS, NTRK1, NTRK2, NTRK3, PDGFRA, PIK3CA, PTEN, RAF1, RET, ROS1, SMO, TP53
**Gene assay for the detection copy number variants**
ALK, AR, CD274, CDKN2A, EGFR, ERBB2, ERBB3, FGFR1, FGFR2, FGFR3, KRAS, MET, PIK3CA, PTEN
**Gene assay for the detection of fusions**
ALK, AR, BRAF, EGFR, ESR1, FGFR1, FGFR2, FGFR3, MET, NRG1, NTRK1, NTRK2, NTRK3, NUTM1, RET, ROS1, RSPO2, RSPO3

**Table 2:** Patient demographics and EBUS procedural details.

	**TBNA**	**TBMC**	**p** **value**
Subjects, *n*	99	104	
Baseline characteristic			
Median age (IQR), years	65.00 (57.00–71.00)	64.00 (56.00–69.75)	0.66
Gender, *n* (%)			
Male	64 (64.65)	69 (66.35)	0.80
Female	35 (35.35)	35 (33.65)	
Smoking, *n* (%)			
Yes	33 (33.33)	24 (23.08)	0.09
Former	12 (12.12)	23 (22.12)	
No	54 (54.55)	57 (54.80)	
Baseline target lesion characteristic			
Total target, *n*	134	140	
Type of lesion, *n* (%)			
Peribronchial mass	36 (26.87)	38 (27.14)	0.96
Lymph node	98 (73.13)	102 (72.86)	
Mean peribronchial mass size (±) (mm)	27.49 (12.20)	26.37 (9.97)	0.66
Peribronchial mass location, *n* (%)			
Upper paratrachea	12 (33.33)	3 (7.89)	0.08
Lower paratrachea	8 (22.22)	9 (23.68)	
Hilar	12 (33.33)	18 (47.37)	
Distal segments	4 (11.11)	8 (21.05)	
Median lymph node size (IQR) (mm)	14.4 (10.15–20.13)	13.95 (11.00–18.15)	0.91
Lymph node station, *n* (%)			
2R	3 (3.06)	5 (4.90)	0.06
4R	32 (32.65)	27 (26.47)	
10R	4 (4.08)	4 (3.92)	
11R	1 (1.02)	6 (5.89)	
7	41 (41.84)	37 (36.27)	
2L	0 (0.0)	1 (0.98)	
4L	16 (16.33)	11 (10.78)	
10L	0 (0.0)	1 (0.98)	
11L	1 (1.02)	10 (9.80)	
Lesion heterogeneity, *n* (%)			
Overall	49 (36.57)	45 (32.14)	0.26
Peribronchial mass	26 (53.06)	29 (64.44)	
Lymph nodes	23 (46.94)	16 (35.56)	
Baseline procedural characteristic			
Sedation, *n* (%)			
Conscious sedation	78 (78.79)	70 (67.31)	0.07
TIVA	21 (21.21)	34 (32.69)	
Median procedural duration (IQR) (minutes)	32 (27–37)	22 (18–32)	< 0.001
Needle size, *n* (%)			
19 gauge	1 (1.01)	—	
21 gauge	9 (9.09)	14 (13.46)	
22 gauge	70 (70.71)	90 (86.54)	
25 gauge	19 (19.19)	—	
Needle suction, *n* (%)			
Capillary pull	16 (16.16)	—	
5cmH_2_0	57 (57.58)	—	
10cmH_2_0	26 (26.26)	—	
Median TBNA passes (IQR), pass	4.00 (4.00–5.25)		
Median TBMC passes (IQR), pass	4 (3–4)		
Median total TBMC cryoactivation time (IQR) (seconds)	24 (18–32)		
Median average TBMC cryoactivation time (IQR) (seconds)	6 (6-8)		
Complication, *n* (%)			
No	65 (65.65)	71 (68.26)	0.48
Mild bleeding	21 (21.21)	23 (22.11)	
Moderate bleeding	3 (3.03)	3 (2.88)	
Desaturation	10 (10.10)	6 (5.76)	
Pneumomediastinum	0	1 (1.00)	
Pneumothorax	0	0	
Mediastinitis	0	0	

Abbreviations: TBMC, transbronchial mediastinal cryobiopsy; TBNA, transbronchial needle aspiration; TIVA, total intravenous anesthesia.

**Table 3:** Diagnostic yield of EBUS-TBNA versus EBUS-TBMC.

		**TBNA**	**TBMC**	**p** **value**
*Diagnostic yield*				
Overall diagnostic yield, *n* (%)	Yes	67 (67.68)	94 (90.38)	< 0.001
	No	32 (32.32)	10 (9.62)	

Common tumour, *n* (%)	Total	47 (47.47)	47 (45.19)	0.75
	NSCLC	42 (89.36)	41 (87.23)	
	SCLC	5 (10.64)	6 (12.77)	

Other metastatic tumour and lymphoma, *n* (%)	Total	8 (8.08)	22 (21.15)	0.006
	Neuroendocrine tumour	0	1 (4.55)	
	Metastatic carcinoma	2 (25.00)	0	
	Meningioma	1 (12.50)	0	
	Neuroblastoma	0	2 (9.09)	
	Neurofibroma	0	2 (9.09)	
	Breast carcinoma	2 (25.00)	4 (18.18)	
	Thymoma	1 (12.50)	0	
	Thymic carcinoma	0	2 (9.09)	
	Hepatocellular carcinoma	0	3 (13.64)	
	Colorectal carcinoma	0	6 (27.27)	
	Prostate carcinoma	0	1 (4.55)	
	Renal cell carcinoma	1 (12.50)	0	
	Diffuse large B-cell lymphoma	1 (12.50)	1 (4.55)	

Benign disorder, *n* (%)	Total	12 (12.12)	25 (24.04)	0.028
	Reactive lymph node	6 (50.0)	5 (20.00)	
	Sarcoidosis	1 (8.33)	4 (16.00)	
	Tuberculosis	5 (41.67)	16 (64.00)	

*Heterogenous lesion*				
Overall diagnostic yield, *n* (%)	Total, *n*	36	39	0.011
	Yes	25 (69.4)	36 (92.3)	
	No	11 (30.6)	3 (7.7)	

Positive diagnostic yield, *n* (%)	NSCLC	16 (64.0)	18 (50.0)	n.a.
	SCLC	3 (12.0)	5 (13.9)	
	Other metastatic tumor	4 (16.0)	7 (19.4)	
	Lymphoma	1 (4.0)	0 (0.0)	
	Sarcoidosis	0 (0.0)	0 (0.0)	
	Tuberculosis	1 (4.0)	6 (16.7)	

*Tumour cell content*				
Median tumour cell content (IQR) (%)	Overall	30 (20–35)	65 (50–75)	0.001
Mean tumour cell content (±) (%)	Nonheterogenous lesion	25.38 (1.94)	58.91 (3.30)	0.001
	Heterogenous lesion	28.75 (8.66)	66.18 (17.28)	0.001

*Molecular analysis*				
Number of cases, *n* (%)	NGS	26 (61.90)	35 (85.36)	0.035
	Limited panel^a^	9 (21.43)	6 (14.63)	
	*EGFR* mutation	4 (9.52)	0 (0.0)	
	Not adequate	3 (7.14)	0 (0.0)	

Abbreviations: n.a., not available; NSCLC, non-small-cell lung carcinoma; SCLC, small-cell lung carcinoma; TBMC, transbronchial mediastinal cryobiopsy; TBNA, transbronchial needle aspiration.

^a^Limited panel: EGFR, ALK and ROS-1 alterations and PD-L1.

## Data Availability

The data that support the findings of this study are available from the corresponding author upon reasonable request.

## Supporting Information

Supporting Information 1 Table S1: Diagnosis for inconclusive cases of EBUS-TBNA and EBUS-TBMC⁣^∗^. NSCLC: non-small-cell lung carcinoma; SCLC: small-cell lung carcinoma; TBNA: transbronchial needle aspiration; TBMC: transbronchial mediastinal cryobiopsy. ⁣^∗^Diagnosis obtained from other modalities including transbronchial lung biopsy, computed tomography-guided biopsy, mediastinoscopy biopsy and video-assisted thoracosurgery biopsy.

Supporting Information 2 Figure S1: Molecular analysis of specimen from EBUS-TBNA versus EBUS-TBMC. TBNA: transbronchial needle aspiration; TBMC: transbronchial mediastinal cryobiopsy.

Supporting Information 3 Table S2: Pilot study for lesion heterogeneity. HU: Hounsfield units; LN: lymph node.
