# Supplementary material for: Performance of Endobronchial Ultrasound-Guided Cryobiopsy in Diagnosing Thoracic Disorders and Its Role in Next-Generation Sequencing for Non-Small-Cell Lung Cancer
Source: Pulm Med. 2025 Aug 28;2025:3522554. doi: 10.1155/pm/3522554 (PMC12411052; doi:10.1155/pm/3522554)
Supplement: Supporting Information 1 — Table S1: Diagnosis for inconclusive cases of EBUS-TBNA and EBUS-TBMC⁣∗. NSCLC: non-small-cell lung carcinoma; SCLC: small-cell lung carcinoma; TBNA: transbronchial needle aspiration; TBMC: transbronchial mediastinal cryobiopsy. ⁣∗Diagnosis obtained from other modalities including transbronchial lung biopsy, computed tomography-guided biopsy, mediastinoscopy biopsy and video-assisted thoracosurgery biopsy. [file 3522554.f1.docx]

Supplementary file 1

Diagnosis for inconclusive cases of EBUS-TBNA and EBUS-TBMC*

|  | | TBNA | TBMC |
| --- | --- | --- | --- |
| **Diagnosis** | | | |
| Total, *n* | | 32 | 10 |
| Number of cases, *n (%)* | NSCLC | 15 (46.88) | 4 (40.00) |
|  | SCLC | 2 (6.25) | 0 |
|  | Other metastatic tumour | 3 (9.37) | 1 (10.00) |
|  | Lymphoma | 1 (3.13) | 0 |
|  | Sarcoidosis | 1 (3.13) | 0 |
|  | Tuberculosis | 3 (9.37) | 0 |
|  | Reactive lymph node | 3 (9.37) | 2 (20.00) |
| **Lost to follow up** | | | |
| Number of cases, *n (%)* | | 4 (12.50) | 3 (30.00) |

NSCLC: Non-small cell lung carcinoma; SCLC: Small cell lung carcinoma; TBNA: Transbronchial needle aspiration; TBMC: Transbronchial mediastinal cryobiopsy.

*Diagnosis obtained from other modalities including transbronchial lung biopsy, Computed tomography-guided biopsy, mediastinoscopy biopsy and video-assisted thoracosurgery biopsy
