# Supplementary material for: Performance of Endobronchial Ultrasound-Guided Cryobiopsy in Diagnosing Thoracic Disorders and Its Role in Next-Generation Sequencing for Non-Small-Cell Lung Cancer
Source: Pulm Med. 2025 Aug 28;2025:3522554. doi: 10.1155/pm/3522554 (PMC12411052; doi:10.1155/pm/3522554)
Supplement: Supporting Information 2 — Figure S1: Molecular analysis of specimen from EBUS-TBNA versus EBUS-TBMC. TBNA: transbronchial needle aspiration; TBMC: transbronchial mediastinal cryobiopsy. [file 3522554.f2.docx]

Supplementary file 2

Molecular analysis of specimen from EBUS-TBNA versus EBUS-TBMC


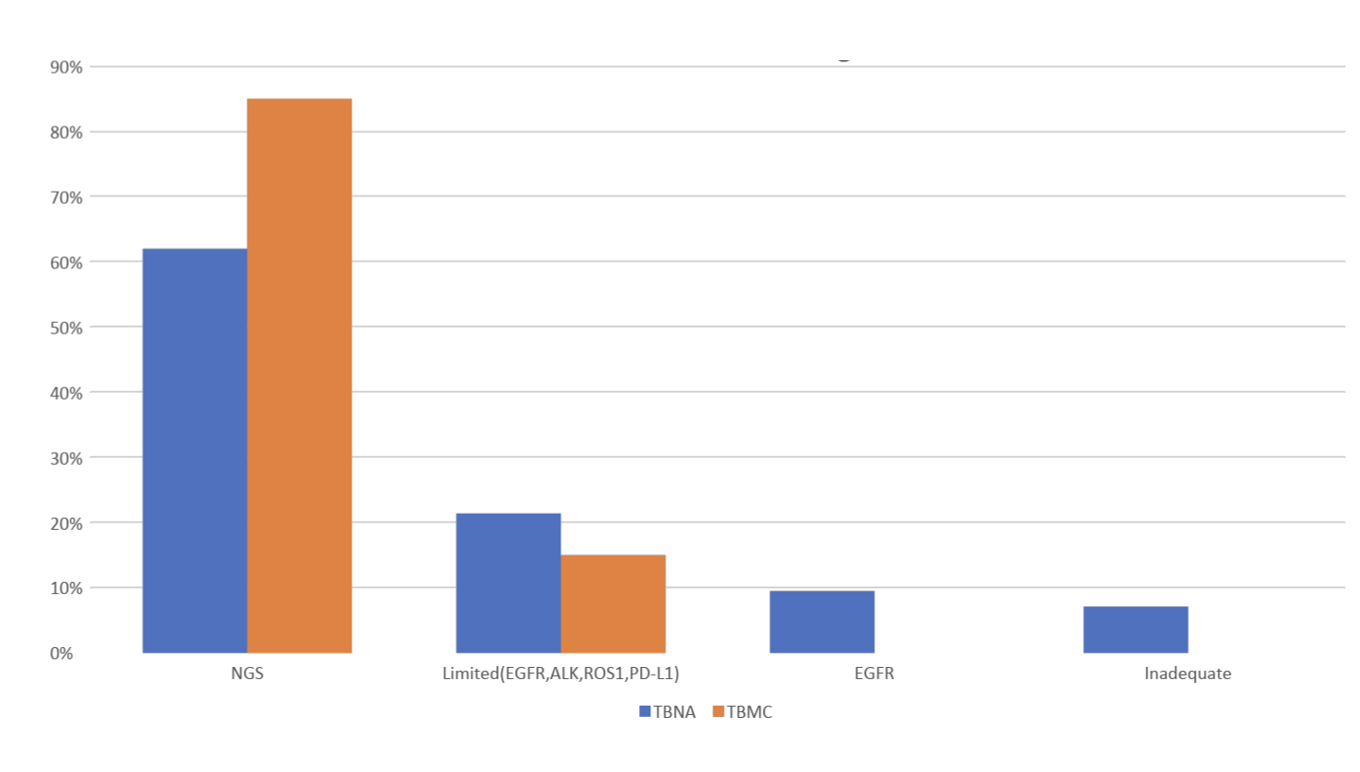


61.90%

7.14%

9.52%

14.63%

21.43%

85.36%

TBNA: Transbronchial needle aspiration; TBMC: Transbronchial mediastinal cryobiopsy
