# Supplementary material for: Performance of Endobronchial Ultrasound-Guided Cryobiopsy in Diagnosing Thoracic Disorders and Its Role in Next-Generation Sequencing for Non-Small-Cell Lung Cancer
Source: Pulm Med. 2025 Aug 28;2025:3522554. doi: 10.1155/pm/3522554 (PMC12411052; doi:10.1155/pm/3522554)
Supplement: Supporting Information 3 — Table S2: Pilot study for lesion heterogeneity. HU: Hounsfield units; LN: lymph node. [file 3522554.f3.docx]

Supplementary 3: Pilot study for lesion heterogeneity

|  |  | Radiologist 1 | | Radiologist 2 | |
| --- | --- | --- | --- | --- | --- |
| Patient | Targeted lesion | Heterogenous | Difference HU | Heterogenous | Difference  HU |
| 1 | 10R LN | No | 17 | No | 13 |
| 2 | 4L LN | No | 9 | No | 11 |
| 3 | 7 LN | Yes | 25 | Yes | 32 |
| 4 | 4R LN | No | 12 | No | 16 |
| 5 | 7 LN | No | 3 | No | 5 |
| 6 | 7 LN | Yes | 35 | Yes | 43 |
| 7 | 7 LN | Yes | 48 | Yes | 37 |
| 8 | Right Hilar mass | Yes | 33 | Yes | 42 |
| 9 | 7 LN | No | 3 | No | 7 |
| 10 | Left hilar mass | Yes | 65 | Yes | 52 |
| 11 | Right upper paratracheal mass | Yes | 81 | Yes | 75 |
| 12 | Right upper paratracheal mass | Yes | 64 | Yes | 58 |
| 13 | 4R LN | No | 9 | No | 11 |
| 14 | 4R LN | Yes | 34 | Yes | 39 |
| 15 | 7 LN | Yes | 50 | Yes | 57 |
| 16 | Right lower paratracheal mass | Yes | 36 | Yes | 29 |
| 17 | Left hilar 1mass | No | 10 | No | 8 |
| 18 | Left lower paratracheal mass | No | 2 | No | 3 |
| 19 | 11L LN | No | 3 | No | 5 |
| 20 | 7 LN | No | 2 | No | 8 |

HU: Hounsfield Units; LN: Lymph node.

Cohen's kappa (κ) was performed to determine the degree of agreement between the radiologists in categorizing heterogeneity in the targeted lesions, the overall Cohen's kappa (κ) value for the study was 0.905 (95% CI, 0.300 – 0.886), P< 0.001
